# Toward Superior Electrochemical Capacitance with Hierarchically Nanostructured Polypyrrole/MXene Hybrid Hydrogel Modified by Lignosulfonate

**DOI:** 10.1021/acsomega.5c02827

**Published:** 2025-06-30

**Authors:** Zhenzhong Hou, Qinghao Yang, Hai Lu, Ying Li, QiuLi Zhao

**Affiliations:** College of Materials Science and Engineering, 118249 Xi’an University of Science and Technology, Xi’an 710054, China

## Abstract

Although the Ti_3_C_2_ MXene has demonstrated exceptional promise for supercapacitor applications, its practical implementation is limited by its inherent tendency of undergoing restacking and oxidation. Herein, we propose a facile self-assembly strategy of in situ polymerization to construct lignosulfonate (LS)-modified polypyrrole (PPy)/MXene hybrid hydrogels with a hierarchical porous structure. The formed PPy nanoparticles and coating layer effectively prevent MXene restacking and oxidation, while enhancing electrical conductivity and electrochemical activity. Furthermore, LS contributes redox-active quinone groups, further promoting electron and proton storage and exchange. The hierarchical heterostructure provides continuous ion-diffusion pathways and exposes abundant electroactive sites, yielding a remarkable specific capacitance of 663.7 F g^–1^ at 0.2 A g^–1^ with 81.2% capacitance retention and 95.3% Coulombic efficiency after 5000 cycles at 2 A g^–1^. Assembled solid-state symmetric supercapacitors achieve an exceptional energy density of 29.3 W h kg^–1^ at 200 W kg^–1^ and maintain 12.4 W h kg^–1^ at an ultrahigh power density of 3200 W kg^–1^, alongside fairly good cycling stability (87.6% capacitance retention) and a Coulombic efficiency of 94.1% over 5000 cycles. These results highlight the multifunctionality of the LS component within the hybrid hydrogels—simultaneously stabilizing the structure, improving electrical conductivity, generating cross-links, and contributing toward pseudocapacitance—establishing LS-modified PPy/MXene hybrid hydrogels as high-performance electrode materials for advanced supercapacitor applications.

## Graphical abstract

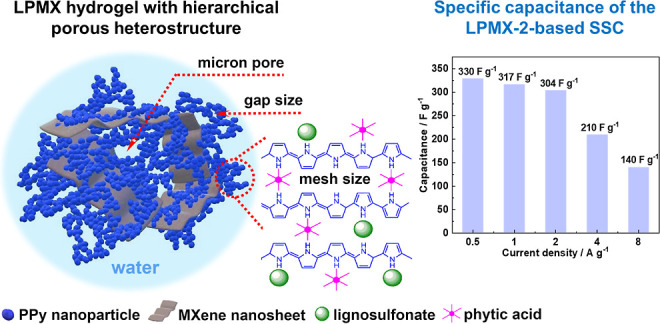

## 1 Introduction

Polymer hydrogels maintain a distinct three-dimensional (3D) network while retaining much water. They are emerging as multifunctional soft materials with substantial theoretical and practical value. Owing to their microstructures that resemble natural tissues, high tunability, and excellent transport properties, polymer hydrogels show considerable application potential in tissue engineering, drug delivery, water purification, and catalyst supports.^1–5^ Typically, conducting polymer hydrogels combine the benefits of hydrogels and organic conductors, offering intrinsic conducting frameworks; efficient transport of charges, ions or molecules; and a large specific surface area resulting from their excellent solid–liquid interface, good electrical characteristics, and easy processability.^6,7^ Consequently, conducting polymer hydrogels are widely applicable in various fields and devices such as energy storage,^8,9^ bioelectronics,^10,11^ sensors,^12,13^ wastewater treatment,^14,15^ and electromagnetic interference shielding.^16,17^

Polypyrrole (PPy) is a well-known conducting polymer commonly used as a supercapacitor electrode material. It suffers from structural pulverization caused by volume expansion and shrinkage during charge–discharge cycles, leading to poor cycling stability.^18^ Developing PPy-based hydrogels with a 3D porous framework may help solve these problems, as such structures allow for multidimensional electron transport and rapid electrolyte diffusion. Shi et al. achieved the in situ polymerization of pyrrole using phytic acid as the gelator and dopant.^19^ The formed single-component PPy hydrogel displayed optimal electrochemical performance: a specific capacitance of approximately 380 F g^–1^ and 93% capacitance retention after 2000 cycles. Therefore, it is critical to rationally design PPy-based composite hydrogels to achieve desired electrochemical performance. Zhang et al. proposed a facile strategy involving a mild hydrothermal reaction to fabricate graphene/PPy composite hydrogel electrodes, achieving a high capacitance of 531 F g^–1^.^20^ A symmetric supercapacitor (SSC) assembled using these electrodes exhibited a high energy density of 15.3 W h kg^–1^ and excellent capacitance retention of 81.7% after 5000 charge–discharge cycles. MXenes, unique two-dimensional (2D) nanomaterials, with high electrical conductivity and hydrophilicity, a large specific surface area, and outstanding electrochemical stability have emerged as leading choices for enhancing PPy electrodes to develop high-performance supercapacitors.^21–23^ For example, Vigneshwaran et al. used MXene/PPy nanocomposites as supercapacitor electrode materials, which achieved a specific capacitance of 474 F g^–1^ at 1 A g^–1^, high-rate performance, and an energy density of 54.4 W h kg^–1^.^24^ Luo et al. prepared PPy nanofiber/MXene films and used them as electrode materials to improve the capacitance of flexible all-solid-state supercapacitors. The optimal supercapacitor demonstrated a specific capacitance of 130.8 F g^–1^ at 0.5 A g^–1^ and excellent cycling stability with 86.8% capacitance retention after 6000 cycles.^25^ In PPy/MXene composite structures, PPy is introduced into MXene layers to elicit a synergistic effect, wherein MXene self-restacking and PPy swelling during charge–discharge cycles are effectively mitigated, yielding optimal composite electrode materials.

To date, a few studies have investigated the use of MXene/PPy hybrid hydrogels in solid-state supercapacitors.^26–28^ In this study, we demonstrate an efficiency and versatile strategy for synthesizing stable lignosulfonate (LS)-modified PPy/MXene hybrid hydrogels via facile in situ polymerization. PPy serves as a blocker to prevent MXene-sheet restacking and as a coating layer to inhibit MXene oxidation. Furthermore, the LS-modified PPy/MXene hybrid hydrogels are employed as active electrode materials to construct symmetric solid-state supercapacitors with good electrochemical performance. The reasons for choosing LS as a modifier in this study are as follows: first, LS promotes the formation of PPy/MXene heterostructure nanocomposites with a 3D hierarchical porous framework while offering morphological controllability. Second, LS contains abundant phenolic hydroxyl groups that can be converted into redox-active hydrazine and hydroquinone structures, enabling the effective storage of abundant electrons and protons, resulting in a high theoretical specific capacitance in the positive potential range.^29,30^ Third, during pyrrole oxidative polymerization, some –OCH_3_ groups from guaiacyl and syringyl units in the LS biomacromolecule can be converted into phenolic hydroxyl groups, generating catechol structures that are similar to the key component in mussel adhesive proteins.^31^ This allows the hybrid hydrogels to easily adhere to the current collector. The exposed heterostructure of the LS-modified PPy/MXene hybrid hydrogels provide numerous active sites for charge and ion transfer, leading to fast ion accessibility and electrolyte penetration. These features contribute to improved electrochemical performances of the hydrogels, making them promising electrode materials for supercapacitors.

## 2 Experimental Section

### 2.1 Materials

The Ti_3_C_2_-MXene was obtained from Jiangsu XFNANO Materials Tech. Co. Ltd. Pyrrole was procured from Sinopharm Chemical Reagent and purified via vacuum distillation before use. Ammonium persulfate (APS; analytical reagent grade), phytic acid (50 wt % aqueous solution), and sodium lignosulfonate (LS) were provided by Aladdin Chemistry Co., Ltd. Carbon cloth, used as a current collector, was obtained from Jinghong New Energy Materials Co., Ltd. All reagents except pyrrole were used as received without further purification. Deionized water from a Millipore Milli-Q system (18.2 MΩ) was used throughout the experiments.

### 2.2 Synthesis

Solution A was prepared by mixing 4 mL of a 0.5 M HCl solution, 0.3 mL of pyrrole, 2 mL of phytic acid, 2 mL of the MXene aqueous solution (5 mg mL^–1^), and a certain amount of LS. Solution B was prepared by dissolving 0.33 g of APS in 1 mL of a 0.5 M HCl solution. Solution A underwent ultrasound treatment for 15 min, generating a homogeneous dispersion. Following this, solutions A and B were rapidly mixed with vigorous stirring for 20 s. The mixture gradually changed from the liquid to gel state within 6 min, and was stored at 0 °C–3 °C for 6 h. After soaking in deionized water for 48 h to remove residual reagents, LS-modified PPy/MXene hybrid hydrogels were obtained. The as-prepared hybrid hydrogels with LS dosages of 5%, 10%, 15% and 20% (mass ratio of LS to pyrrole) are herein referred to as LPMX-1, LPMX-2, LPMX-3 and LPMX-4, respectively. The detailed compositions and electrical conductivities of the LPMX samples are presented in Table S1 of Supporting Information.

### 2.3 Characterization

The micromorphologies of the prepared hydrogel samples were examined using scanning electron microscopy (SEM; ZEISS Sigma 360, Germany) and transmission electron microscopy (TEM; FEI Talos F200X, the USA). Energy-dispersive X-ray spectroscopy (EDS) mapping was employed to investigate the elemental distribution on the sample surfaces. Fourier transform infrared (FTIR) spectroscopy (Bruker Equinox 55 spectrophotometer) and Raman spectroscopy (LabRam Aramis, 532 nm) were employed to analyze the absorption characteristics and structural compositions of the samples. X-ray photoelectron spectroscopy (XPS; Thermo Scientific K-Alpha, USA) was employed to determine the surface elemental valence states and chemical compositions of the samples. X-ray diffraction (XRD; Bruker D8 Advance, Cu–Kα radiation wavelength of 0.154 nm) was employed to analyze the crystal structures of the hydrogel samples. The Brunauer–Emmett–Teller (BET) specific surface areas and total pore volumes of the samples were determined using a surface analyzer (Micromeritics ASAP-2460). The electrical conductivities of the samples were measured using an SDY-4 digital four-probe instrument (Wuhan Corrtest, China).

### 2.4 Electrochemical Measurements

The prepared hybrid hydrogel electrodes were characterized via cyclic voltammetry (CV), galvanostatic charge/discharge (GCD) and electrochemical impedance spectroscopy (EIS) in a 1 M H_2_SO_4_ solution at room temperature using an electrochemical workstation (CS 350, Corrtest Corporation, China) with a conventional three-electrode system. A saturated calomel electrode (SCE) and a platinum plate (ca. 1 cm^2^) were used as the reference and counter electrodes, respectively. The working electrode was prepared as follows: first, a carbon cloth (ca. 1 cm^2^) was immersed in a mixture of H_2_SO_4_ and HNO_3_ (volume ratio = 2/1) to activate at 100 °C for 5 h. It was then washed several times with ethanol and deionized water, followed by drying at 60 °C. Second, the activated carbon cloth was immersed in solution A for 60 s. Subsequently, solution B was directly dropped onto the carbon cloth for self-assembly and in situ polymerization at 0 °C. Finally, this polymerization process was repeated five times to produce the hybrid hydrogel electrodes, which were stored for 12 h at room temperature before use. The mass loading of the active material in the electrodes was ∼2.6 mg cm^–2^. For comparison, a PPy/MXene hybrid hydrogel (referred to as PMX) electrode was also prepared under the same conditions but without LS. Furthermore, a solid-state SSC was assembled using two identical hybrid hydrogel electrodes, a PVA/H_2_SO_4_ gel electrolyte, and a paper separator. Before assembly, the hybrid hydrogel electrodes were immersed in the gel electrolyte solution for 2 h to ensure full electrolyte penetration.

The mass specific capacitance (C, F g^–1^), energy density (E, W h kg^–1^) and power density (P, W kg^–1^) were calculated from the GCD curves using the following equations

1 $$C=\frac{I\times \Delta t}{m\times \Delta V}$$

2 $$E=\frac{C\times {\left(\Delta V\right)}^{2}}{2\times 3.6}$$

3 $$P=\frac{E}{\Delta t}\times 3600$$

where *I*, m, Δ*t* and Δ*V* represent the discharge current (A), total mass of the active material within the electrodes (g), discharge time (s), and potential range (V), respectively.

## 3 Results and Discussion

### 3.1 Morphological Characterization

Figures 1 and S1 (Supporting Information) collectively present the structural blueprints of the LPMX hydrogels, showing schematic illustrations of their hierarchical architectures alongside macroscopic images. Due to the combined gelation effects of phytic acid and LS, MXene nanosheets self-assembled with in situ generated PPy nanoparticles to generate a hierarchical porous structure. Strong interfacial interaction ensured the immobilization of the PPy nanoparticles across MXene surfaces. SEM and TEM results (Figures 2 and 3, respectively) confirmed two-level porosity in the LPMX matrix. Primary pores resulted from nanoscale voids named gap sizes between interconnected PPy nanoparticles, and secondary pores were micropores, as indicated by arrows in Figures 1, 2, and 3. This hierarchical architecture created an optimized 3D conduction network, exhibiting a large electroactive surface area for enhanced charge storage, open channels for accelerated ion diffusion, and interconnected charge transport pathways for improved rate capability.

**Figure 1:**
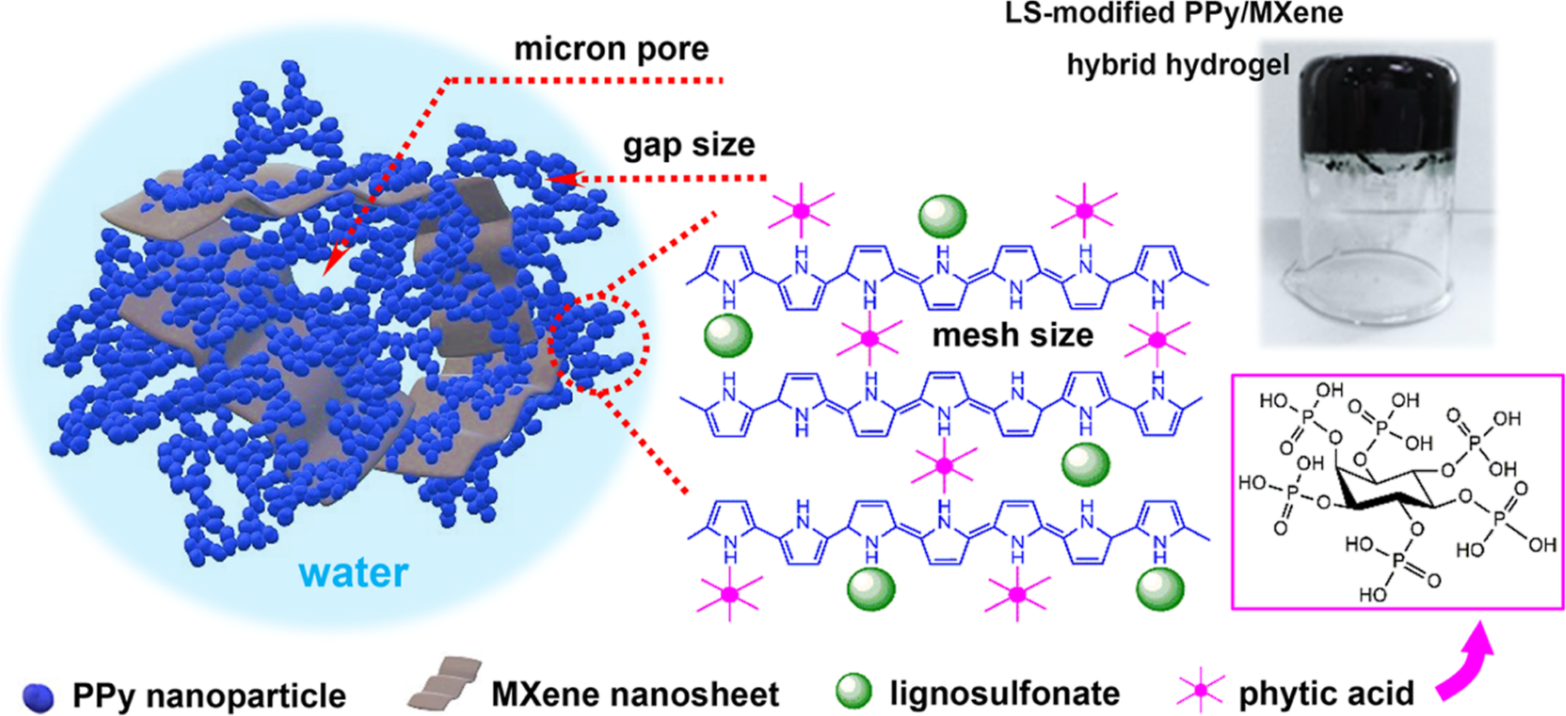
Schematic of the hierarchical porous structure of a representative LPMX hydrogel alongside its macroscopic image.

**Figure 2:**
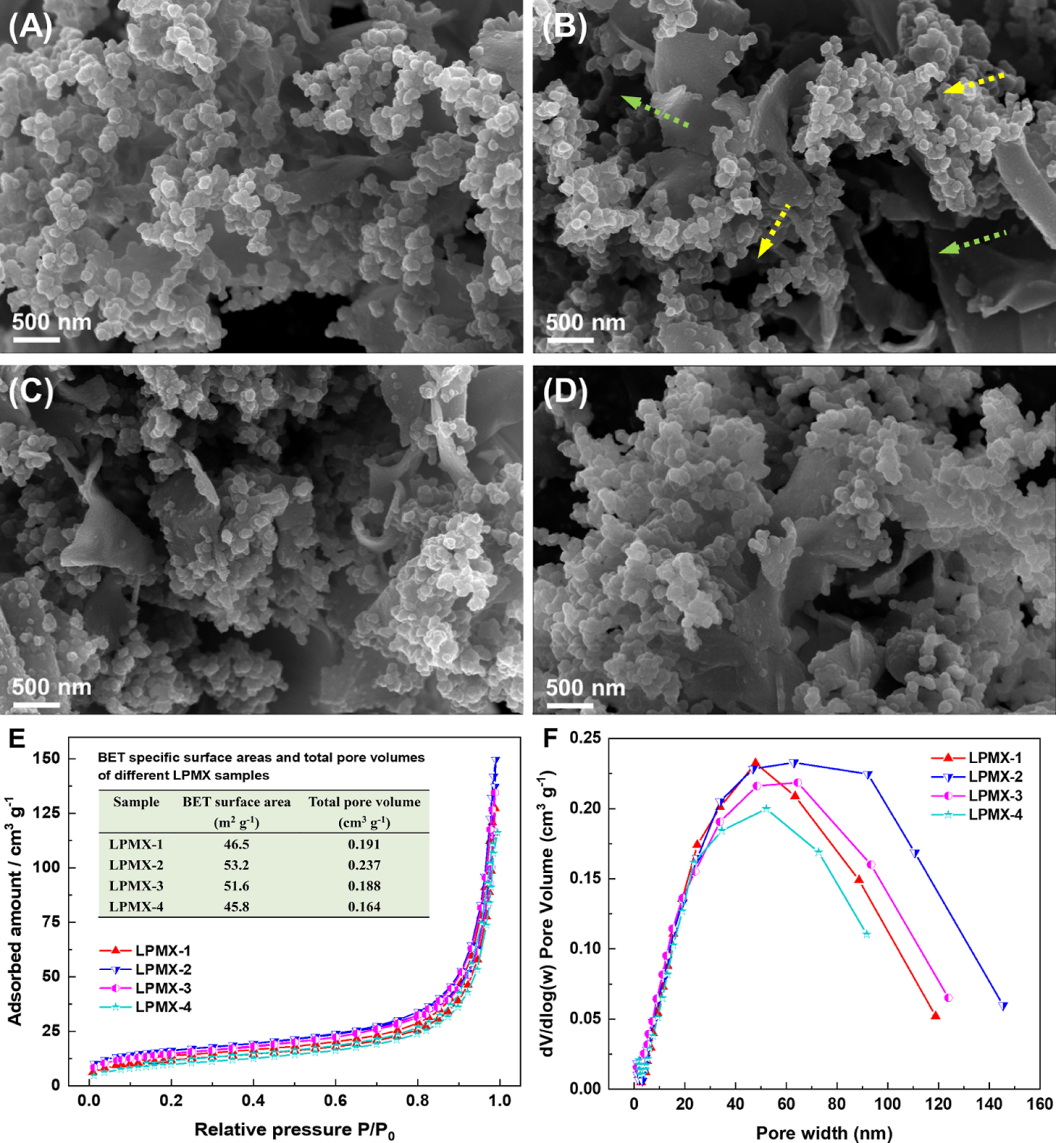
SEM images of the LPMX samples. (A) LPMX-1, (B) LPMX-2, (C) LPMX-3, (D) LPMX-4, (E) N_2_ adsorption–desorption isotherms of the LPMX samples; inset: detailed presentation of the BET specific surface areas and total pore volumes, and (F) pore-size distribution of the LPMX samples.

**Figure 3:**
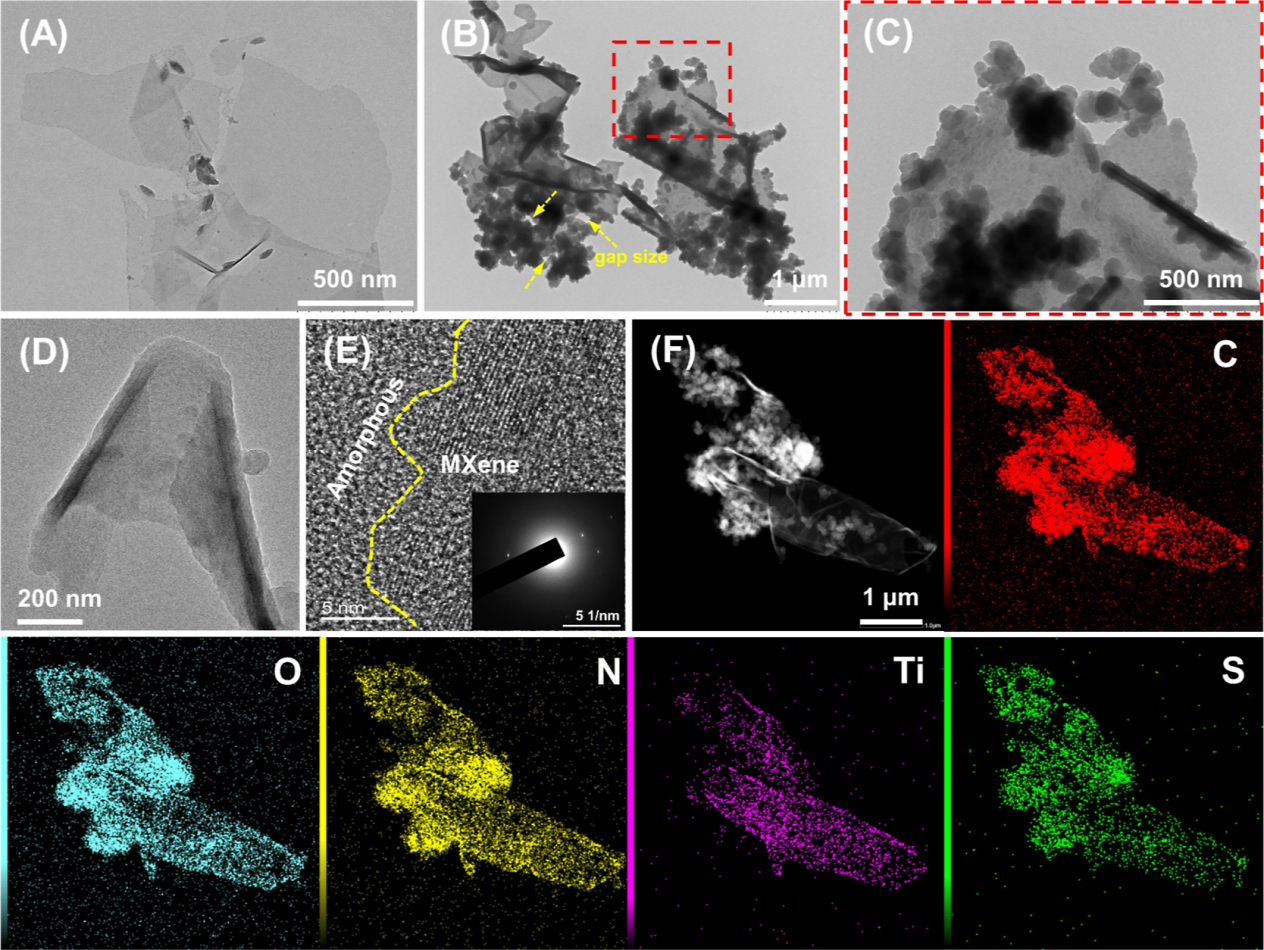
TEM images of (A) the MXene and (B,C) LPMX-2, (D) TEM image of the MXene@PPy structure in LPMX-2, (E) HRTEM image and SAED pattern of the MXene@PPy structure, and (F) HAADF image of LPMX-2 and the corresponding elemental mapping spectra.

Phytic acid and LS played crucial roles in the gelation, nanostructure formation, and electrochemical optimization of the LPMX composites. Phytic acid effectively doped the PPy backbones to enhance electrical conductivity, and its hexaphosphoric architecture established cross-linking points through electrostatic interactions with multiple protonated PPy chains (Figure 1). This molecular bridging effect simultaneously facilitated 3D hydrogel assembly and enhanced surface hydrophilicity via residual phosphate groups, improving the electrolyte wettability of the samples. Meanwhile, LS served as a bifunctional modulator, regulating dopant distribution and sample morphology. The electrical conductivity profile of the samples followed a characteristic volcano trend with increasing LS content (from 5 to 20 wt %), reaching a peak value of 36.8 S cm^–1^ at an optimal loading of 10 wt % (Table S1, Supporting Information). At this content, LS enabled extended π-conjugation within PPy through ordered chain alignment while maintaining sufficient charge carrier mobility. As the LS content increased, the steric hindrance effect became increasingly pronounced, disrupting π-conjugation along PPy chains.^32,33^ Combined with the inherent electrical insulation capability of LS, these factors substantially reduced the conductivity of the LPMX composites.

Figure 2 shows LS-mediated variation in the microstructural evolution of the LPMX samples. All specimens exhibited heterostructures of interconnected PPy nanoparticles and MXene nanosheets stabilized by synergistic interfacial binding mechanisms, including chemical bonding and physical mixing. MXene nanosheets offered additional nucleophilic reactive sites for pyrrole monomers via terminal functional groups, with the PPy skeleton binding closely to the MXene nanosheet surface through electrostatic interactions, hydrogen bonds, and covalent bonds. Additionally, some PPy molecules interacted with the MXene via van der Waals forces and π–π stacking, which were physical processes. Some typical micropores (green arrows) and gap sizes (yellow arrows) are shown in Figure 2B. High-resolution imaging revealed that PPy nanoparticles nucleated preferentially on MXene surfaces and functioned as intrinsic spacers that mitigated the restacking of MXene nanosheets and generated oxidation-resistant composite interfaces. LS played a key role in the formation and size control of PPy nanoparticles. As the LS content increased from 5% to 20%, the diameter of the formed PPy particles gradually decreased from ∼120 nm to <80 nm. Excessive size reduction caused severe PPy nanoparticle aggregation, disrupting the uniformity of the PPy/MXene heterostructure. Figure 2E shows the N_2_ adsorption–desorption isotherms of the LPMX samples, revealing typical type-IV behavior. The tightly clustered adsorption–desorption profiles of the LPMX samples indicate comparable BET surface areas and pore volumes, as summarized in the inset data panel of Figure 2E. As the LS content increased, PPy nanoparticles were more easily inserted between the MXene layers, expanding the specific surface area and pore volume of the LPMX samples. However, excessive LS leaded to smaller PPy nanoparticles, which tended to aggregate and wrap around the multilayer MXene surface, thereby reducing the specific surface area and pore volume of the samples. The LPMX-2 sample demonstrated the best performance, achieving the highest specific surface area (53.2 m^2^ g^–1^) and pore volume (0.237 cm^3^ g^–1^). This superiority stemmed from the effective intercalation of the PPy nanoparticles between the MXene nanosheets, which inhibited MXene nanosheet restacking and generated numerous hierarchical voids in the interconnected network. The pore-size distribution of the LPMX samples (Figure 2F) further confirmed the presence of hierarchical pores. The pore sizes were primarily distributed between 30 and 110 nm, corresponding to mesopores (2–50 nm) and macropores (>50 nm), respectively. These structural features increased the accessible surface area and porosity, facilitated rapid ion and electrolyte transport kinetics, and optimized the electrode–electrolyte interfacial contact. These synergistic effects made LPMX-2 a promising candidate for supercapacitor electrode material with excellent electrochemical performance.

TEM images (Figure 3) revealed the pleated morphology of ultrathin MXene nanosheets and the hierarchical porous structure of LPMX-2. From Figure 3B,C, it is evident that numerous PPy nanoparticles with sizes of <100 nm were loaded onto the MXene nanosheets, forming abundant voids with gap sizes ranging from tens to over a hundred nanometers long, as indicated by arrows in Figure 3B. These gap sizes and micropores constituted the hierarchical porous structure. Furthermore, Figure 3D shows a MXene nanosheet coated with PPy nanoparticles, forming a “core@shell”-like heterostructure. The HRTEM image of the MXene@PPy structure (Figure 3E) was divided into crystalline and amorphous regions, corresponding to the MXene and PPy, respectively, further indicating the successful introduction of PPy. However, the corresponding SAED inset does not exhibit the expected diffraction ring characteristics. The possible reason was that the amorphous PPy coating on MXene surfaces severely interfered with SAED pattern. Figure 3F shows the TEM image of LPMX-2 along with elemental mapping results of C, O, N, Ti and S for the sample. These elements were uniformly distributed in the composite matrix, confirming the homogeneous dispersion of various components. Moreover, the EDS pattern and elements ratios are showed in Figure S2 (Supporting Information). After estimation, the weight percentages of PPy and MXene in dehydrated LPMX-2 sample are about 87% and 2%, respectively.

### 3.2 Structural Characterization

The FTIR spectra of the MXene, PPy and LPMX samples are shown in Figure 4A. In the MXene spectrum, the two strong peaks at 3446 and 1645 cm^–1^ were attributed to –OH and = O groups, respectively, and weak peaks at 1388, 1240, and 1065 cm^–1^ corresponded to –F and other surface terminal groups.^34,35^ In the PPy spectrum, the characteristic stretching vibrations of pyrrole rings were observed at 1543 and 1455 cm^–1^,^36^ and other PPy-specific bands in the 1400–750 cm^–1^ region were attributed to C–N stretching, N–H/C–H deformation, and C–C bending vibrations.^37,38^ The LPMX spectra exhibited characteristic peaks of the MXene and PPy, confirming successful hybrid hydrogel synthesis. Additionally, LS generated peaks at 1046 and 670 cm^–1^ in the LPMX spectra, which corresponded to S=O bond stretching vibrations. A closer inspection of the LPMX spectra revealed potential interfacial interactions between the PPy nanoparticles and MXene nanosheets in the LPMX samples, as evidenced by subtle shifts in some peaks: the 1385 cm^–1^ peak shifted to a lower wavenumber and the 1552, 1190, and 912 cm^–1^ peaks shifted to higher wavenumbers compared with their positions in the spectra of the individual components.

**Figure 4:**
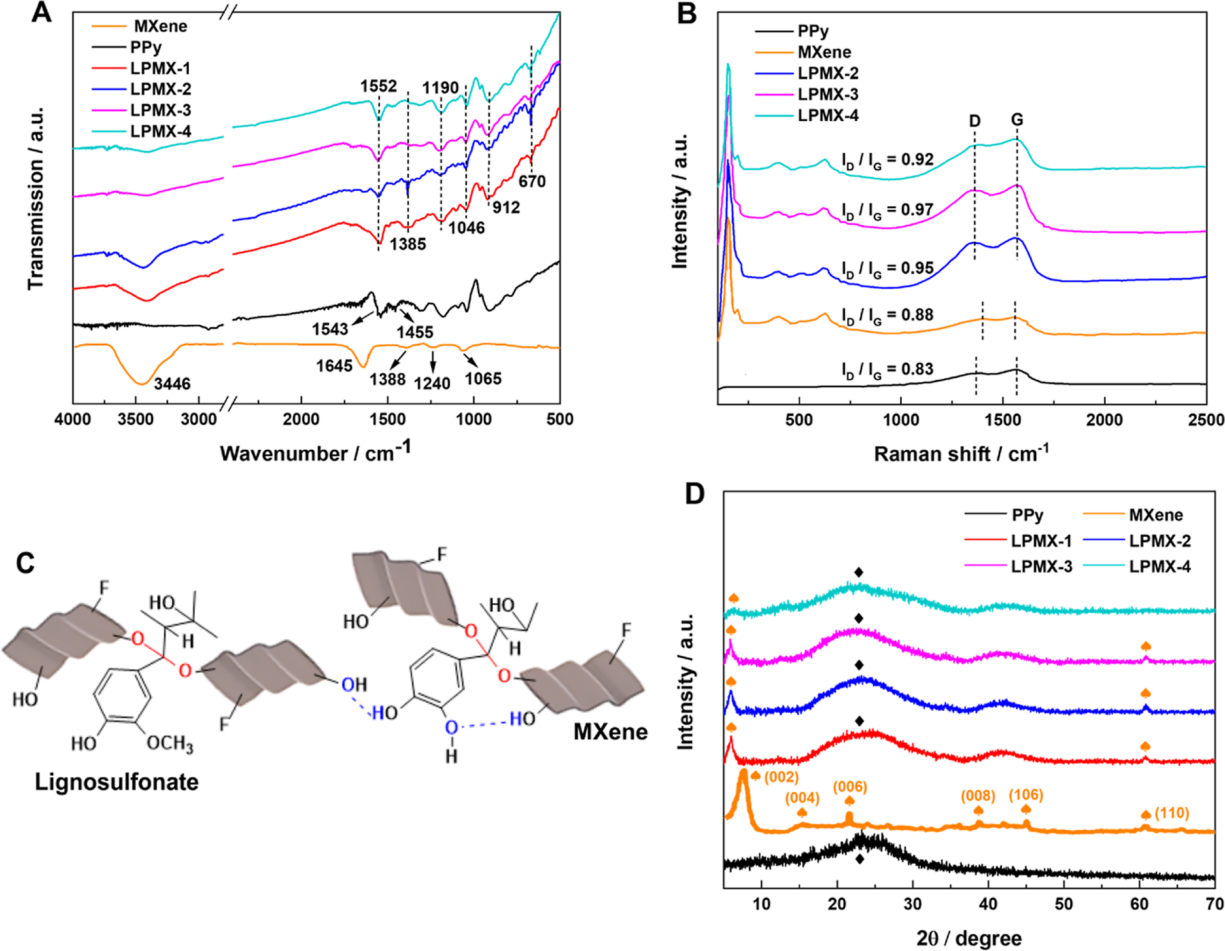
(A) FTIR and (B) Raman spectra of PPy, the MXene, and the LPMX samples, (C) schematic of LS modifying the MXene surface through chemical bonds, and (D) XRD patterns of PPy, the MXene, and the LPMX samples.

As a complementary technique, Raman spectroscopy provided additional structural insights regarding the LPMX samples (Figure 4B). The MXene spectrum displayed three distinctive peaks at 155, 403, and 627 cm^–1^, corresponding to the vibrations of nonstoichiometric carbon.^35,39^ In the PPy spectrum, the peaks at 1369 and 1568 cm^–1^ were attributed to C–C/C–N stretching and the π-conjugated structure, respectively.^38,40^ The LPMX spectra showed hybrid peaks from PPy and MXene, confirming the successful decoration of the MXene nanosheets by the PPy nanoparticles. Notably, two prominent peaks at ∼1352 and 1570 cm^–1^ (labeled D and G, respectively) in the LPMX spectra exhibited slight shifts compared to their positions in the MXene and PPy spectra. This suggested strong interactions between PPy and the MXene, which aligned with the FTIR spectroscopy results. The D and G peaks corresponded to disordered carbon and sp^2^-hybridized graphitic carbon, respectively.^41^ A high intensity ratio of the D and G peaks (*I*_D_/*I*_G_) indicates a disordered structure. The LPMX samples showed higher *I*_D_/*I*_G_ values than PPy and the MXene, suggesting the presence of more edges and defects, which enhanced the electrochemical activity of the samples.^42^ Furthermore, LS decorated the MXene surface through C–O (red) and hydrogen (blue) bonds, as shown in Figure 4C. These C–O bonds formed from chemical interactions between LS and the MXene surface, where sulfonic acid groups at the α position were substituted by C–O groups.^43^ LS acted as a stabilizer, dopant, modifier, and cross-linker, serving as a versatile additive. It effectively linked the MXene nanosheets and PPy nanoparticles through covalent and noncovalent bonding, facilitating the formation of an interconnected microstructure with enhanced mechanical stability and charge-transfer properties.

XRD measurements (Figure 4D) were performed to investigate the crystalline structures and phase compositions of the LPMX samples, PPy, and the MXene. The MXene spectrum revealed prominent characteristic peaks corresponding to the (004), (006), (008), (106), and (110) planes, consistent with its well-defined 2D layered structure.^44^ Specifically, the sharp diffraction peak at 7.6° corresponded to a layer spacing of 1.162 nm, which pertained to the (002) crystal plane of the MXene. PPy exhibited a broad peak centered at ∼23°, indicating its predominantly amorphous nature. The characteristic peaks of the MXene and PPy were observed simultaneously in the LPMX samples. Moreover, the (002) characteristic peak of the LPMX samples shifted to 5.9°, which corresponded to an increase of 0.335 nm in the MXene interlayer spacing. Notably, the (002) characteristic peak in the LPMX-4 spectrum shifted to 6.3° and became weak, and its (110) characteristic peak nearly disappeared, indicating worse intercalation of the PPy nanoparticles between the MXene nanosheets in the LPMX-4 matrix compared to those of the other LPMX samples, which further confirmed the N_2_ adsorption–desorption results. This finding strongly supported the decisive role of LS-modified PPy nanoparticles in expanding the MXene interlayer spacing.

The chemical composition and valence states of the LPMX-2 sample were further analyzed using high-resolution XPS spectra, as presented in Figure 5. The survey spectrum included five peaks assigned to C, N, O, Ti, and S, with the C 1s, N 1s, O 1s, Ti 2p, and S 2p core-level spectra presented in Figure 5B–F. The C 1s spectrum was fitted using three peaks at 284.8, 286.2, and 289 eV, which were attributed to C–C, C–N, and C–O/C=O bonds, respectively.^25^ The N 1s spectrum showed four peaks at 397.8, 399.6, 400.3, and 402 eV, corresponding to N–Ti, –NH–, –N^+^H–, and –N = bonds, respectively.^39,45^ The emergence of the N–Ti peak indicated strong interfacial coupling between PPy and the MXene, and the –N^+^H– cation was considered a PPy polaron, which contributed to electrochemical activity and facilitated the pseudocapacitive process. The O 1s spectrum was divided into three peaks at 530.5, 531.1, and 532.5 eV, corresponding to Ti–O bonds, cavity O, and Ti–O–H bonds, respectively.^25^ In the Ti 2p spectrum, four major peaks were observed: Ti–C 2p_3/2_, Ti–O 2p_3/2_, Ti–C 2p_1/2_, and Ti–O 2p_1/2_, which were located at ∼454.9, 459, 460.9, and 464.7 eV, respectively. Moreover, the S 2p spectrum was deconvoluted into two peaks near 168.4 and 170.7 eV, corresponding to –SO_3_^–^ 2p_3/2_ and –S– 2p_3/2_, respectively, providing direct evidence of the presence of the LS component within the LPMX matrix. Overall, XPS results revealed numerous electrochemical active sites and functional groups in the LPMX samples, which contributed to the enhancement of the electrochemical capacity.

**Figure 5:**
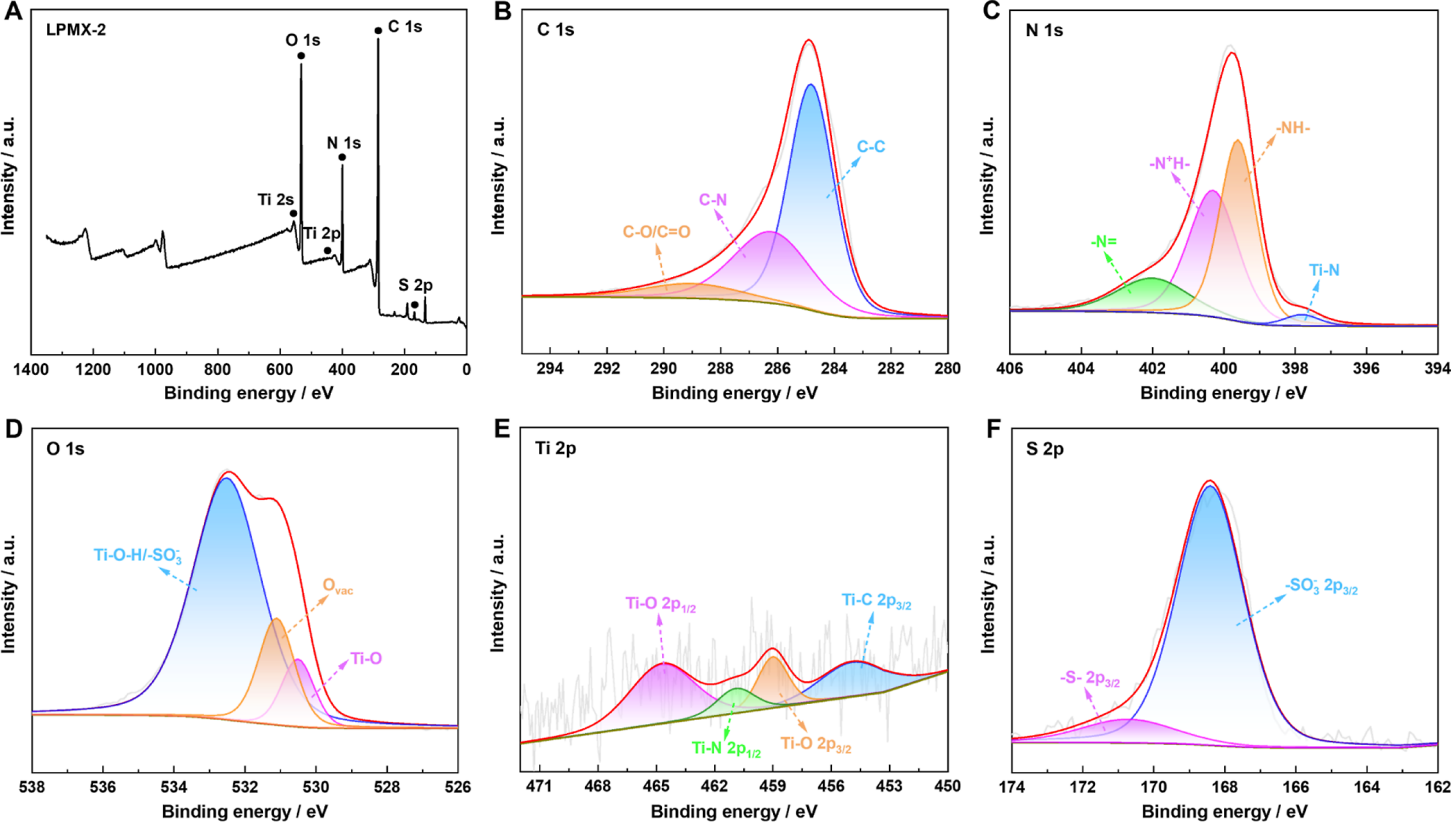
High-resolution XPS spectra of LPMX-2: (A) survey spectrum and (B) C 1s, (C) N 1s, (D) O 1s, (E) Ti 2p, and (F) S 2p spectra.

### 3.3 Electrochemical Characterization

The electrochemical performance of the fabricated PMX and LPMX electrodes was evaluated using a standard three-electrode system with a 1 M H_2_SO_4_ aqueous solution as the electrolyte. Figure 6A shows the CV curves at a scan rate of 2 mV s^–1^, showing similar distorted rectangular shapes, indicating strong pseudocapacitive properties. Notably, the CV curve of LPMX-2 showed the largest loop area and strongest current response, suggesting superior capacitive performance compared to those of the other samples. LPMX-2 outperformed the other samples owing to its larger specific surface area, higher pore volume, greater electrical conductivity, and more number of active sites. Figure 6B shows the GCD curves of the PMX and LPMX electrodes at a current density of 0.2 A g^–1^. The curves showed a deformed, asymmetric triangular shape without a distinct plateau, confirming good pseudocapacitive behavior paralleling the CV results. LPMX-2 had the highest specific capacitance because of its longest discharge duration. The specific capacitances of the PMX, LPMX-1, LPMX-2, LPMX-3, and LPMX-4 at 0.2 A g^–1^ were 300.2, 498.6, 663.7, 355, and 257.5 F g^–1^, respectively, as calculated using eq 1. Figure 6C shows the specific capacitance values of the PMX and LPMX electrodes at current densities of 0.2–10 A g^–1^. LPMX-2 consistently outperformed the other samples, exhibiting the highest specific capacitance across all current densities. Further analysis revealed that LPMX-3 had the highest rate capability, with 51.5% capacitance retention at 10 A g^–1^, followed by LPMX-2 with 46.3% retention. However, PMX showed the lowest rate capability with 21.5% retention. Moreover, as the current density increased, the specific capacitance of all samples decreased, indicating challenges in redox reactions, ion diffusion, and charge adsorption during rapid charge–discharge cycles. These results confirmed that LS markedly improved the electrochemical performance of the LPMX samples.

**Figure 6:**
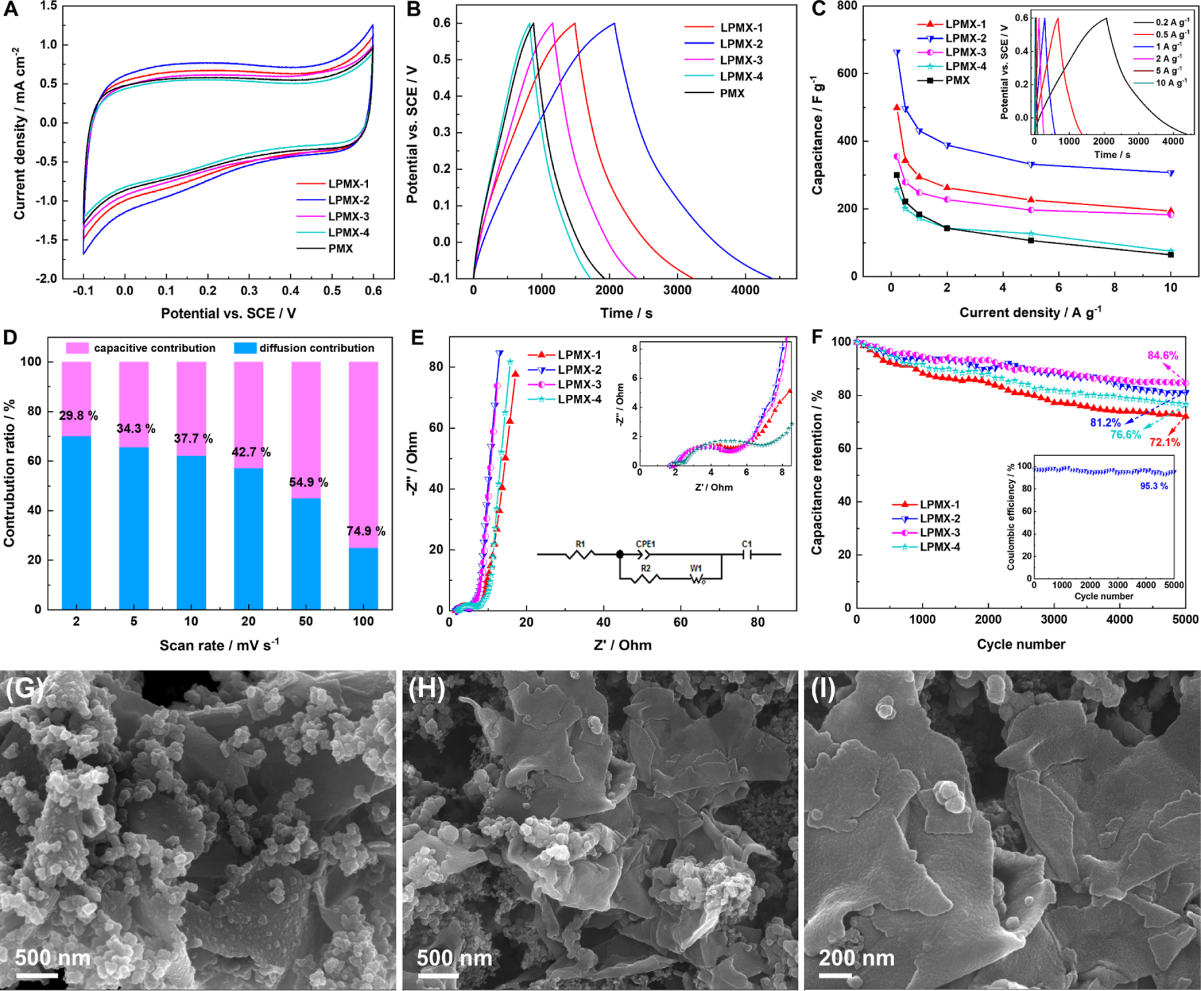
Electrochemical characterization of the LPMX and PMX electrodes, and SEM images of the LPMX-2 electrode material before and after cycling tests. (A) CV curves at 2 mV s^–1^, (B) GCD curves at 0.2 A g^–1^, (C) variation curves of the specific capacitance with the current density; inset: GCD curves of LPMX-2 from 0.2–10 A g^–1^, (D) capacitive contribution ratios of LPMX-2 at various sweep rates, (E) Nyquist plots of the LPMX samples; inset: impedance diagrams at high frequencies and the equivalent circuit, (F) cycling stability of the LPMX samples at 2 A g^–1^; inset: Coulombic efficiency of LPMX-2 at the same condition, (G) LPMX-2 before cycling, and (H–I) LPMX-2 after cycling.

The electrochemical properties of the LPMX samples were dependent on their microstructures. The synergistic effects of phytic acid and LS enabled interconnected PPy nanoparticles to infiltrate the MXene layers, forming a conductive network that prevented MXene nanosheet restacking and oxidation while enhancing electrical conductivity. In addition, the MXene nanosheets self-assembled into another conductive network. Together, these networks created a unique hierarchical porous structure with two energy storage mechanisms. This structure shortened the charge transport path, improved the ion-diffusion rate, and increased the number of exposed active sites, all contributing to enhanced electrochemical performance. Increasing the LS/pyrrole mass ratio resulted in the generation of smaller PPy nanoparticles, which enhanced the porous nanostructure but reduced the electrical conductivity and specific active surface area owing to the intrinsic electrical insulation effect of LS and the agglomeration of PPy nanoparticles. The trade-off between the surface area, porosity, and electrical conductivity of the hybrid hydrogels limited the tunability of the electrochemical performance of the LPMX electrodes. Typically, the capacitive and diffusion-controlled contributions to the total capacitance of electrode materials can be accurately quantified using the following equations^46^

4 $$I=k_{1}v+k_{2}v^{1/2}$$

Eq 4 can be reformulated as

5 $$\frac{I}{v^{1/2}}=k_{1}v^{1/2}+k_{2}$$

here, *k*_1_ and *k*_2_ are constants obtained from CV profiles at different scanning rates. *k*_1_ *v* and ${k_{2}v}^{1/2}$ represent capacitive process and diffusion-controlled reactions, respectively. Figure 6D shows the contribution ratios of capacitive and diffusion-controlled processes for the LPMX-2 electrode at various scan rates. With increasing scan rate, the proportion of the capacitive process gradually increased from 29.8% at 2 mV s^–1^ to 74.9% at 100 mV s^–1^ (as indicated by the magenta shaded area in Figure S3, Supporting Information), confirming the progressively enhanced reaction kinetics of the LPMX-2 electrode.^46^

EIS was employed to investigate the frequency response characteristics of the LPMX electrodes. Figure 6E presents the Nyquist plots of the LPMX electrodes and the corresponding fitted equivalent circuit. Each plot displays a quasi-semicircle in the high-frequency region and a straight line in the low-frequency region. The high-frequency intercept on the real axis represents the equivalent series resistance (*R*_1_), which includes the electrolyte resistance, intrinsic resistance of the active material, and contact resistance between the active material and current collector.^39^ The diameter of the semicircle in the high-frequency region typically corresponds to the charge-transfer resistance (*R*_2_) at the electrode–electrolyte interface. Detailed fitted parameters are provided in Table 1, where CPE_1_ represents the constant phase angle element, *W*_1_ denotes the Warburg impedance, and *C*_1_ is the Faradaic pseudocapacitance.^47^ Notably, the LPMX-2 electrode exhibited the lowest *R*_1_ and *R*_2_ values, indicating improved ion migration within the electrode material and minimal ion-transfer barrier. In addition, the Nyquist plot for LPMX-2 in the low-frequency region showed a notably steep slope, indicating nearly ideal capacitive behavior. Figure 6F illustrates the long-term cycling stability of the LPMX electrodes, with GCD tests performed at 2 A g^–1^ for 5000 cycles. LPMX-2 and LPMX-3 showed retention rates of 81.2% and 84.6%, respectively, demonstrating excellent cycling performance. This performance was attributed to the incorporation of LS and the formation of a complex porous nanostructure, which reduced resistance, increased number of transport channels, provided additional electrochemically active sites, and stabilized the framework. Notably, excessive LS may deteriorate cycling stability. In addition, the Coulombic efficiency of LPMX-2 was maintained at ∼95.3% (Figure 6F inset), the highest among the LPMX samples (Figure S4, Supporting Information), indicating efficient charge–discharge capability. After cycling tests, the PPy coating and nanoparticles partially detached from the surfaces of the MXene layers, and the fractured and restacked MXene nanosheets also appeared (Figure 6G–I), leading to a decrease in the specific capacitance of the LPMX-2 electrode.

**Table 1:** Fitting Data for the Equivalent Circuit Elements

sample	*R*_1_/Ω	CPE_1_/F	*R*_2_/Ω	*W*_1_/Ω	*C*_1_/F
LPMX-1	1.863	0.0421	4.022	0.1953	0.1975
LPMX-2	1.825	0.0373	3.516	0.3120	0.1669
LPMX-3	2.013	0.0037	3.837	0.3037	0.1964
LPMX-4	2.173	0.0064	5.943	0.2974	0.1743
SSC	2.096	0.0025	3.612	0.2828	0.5873

Based on these results, the LPMX-2 electrode was selected to fabricate an SSC and further investigate the capacitance performance. A comparison of the capacitive performances of LPMX-2 in a three-electrode system with those of other recently reported PPy/MXene composites is provided in Table 2.^24,25,44,48–57^ LPMX-2 demonstrated superior specific capacitance and cycling stability, highlighting its exceptional energy storage capacity achieved through multiple self-assembled in situ polymerization processes.

**Table 2:** Comparison of the Capacitive Performance of Various PPy/MXene Composites in a Three-Electrode System

electrode material	electrolyte	specific capacitance/F g^–1^	capacitance retention/cycles	refs
PPy/MXene nanocomposite	1 M H_2_SO_4_	474 (1 A g^–1^)	96% (1 A g^–1^)/10000	^24^
MXene/PPy nanofiber composite film	1 M H_2_SO_4_	437.2 (1 A g^–1^)	79.5% (5 A g^–1^)/6000	^25^
(PPy/MXene)@cotton fiber	1 M H_2_SO_4_	506.6 (1 A g^–1^)	83.3% (3 A g^–1^)/2000	^44^
PPy/MXene composite	0.5 M Na_2_SO_4_	85 (0.2 A g^–1^)	94% (50 mV s^–1^)/1000	^48^
rGO/MXene–PPy film	1 M H_2_SO_4_	408.2 (1 A g^–1^)		^49^
α-NSA doped PPy/MXene composite	2 M KCl	347.6 (5 mV s^–1^)	92% (100 mV s^–1^)/4000	^50^
MXene/PPy-decorated carbon nanofibers	1 M NaCl	425 (0.25 A g^–1^)	78% (1 A g^–1^)/250	^51^
PPy/MXene composite	1 M Na_2_SO_4_	300.8 (1 A g^–1^)		^52^
MXene/dopamine-modified PPy nanofiber composite film	1 M H_2_SO_4_	384.9 (1 A g^–1^)	86.7% (2 A g^–1^)/3000	^53^
MXene/hydrophilic PPy composite film	3 M H_2_SO_4_	369.6 (2 mV s^–1^)		^54^
MOF@PPy@MXene/CNT	3 M KOH	1567.5 (1 A g^–1^)	91.2% (10 A g^–1^)/10000	^55^
PPy/MXene/alginate aerogel	2 M H_2_SO_4_	475 (1 A g^–1^)	81% (10 A g^–1^)/1500	^56^
PPy@MXene/polyurethane fiber	1 M H_2_SO_4_	32 (0.1 A g^–1^)	90.4% (0.3 A g^–1^)/10000	^57^
LPMX-2	1 M H_2_SO_4_	663.7 (0.2 A g^–1^)	81.2% (2 A g^–1^)/5000	this work
		431.2 (1 A g^–1^)		

To further explore the practical applications of the LPMX-2 sample, the sample was integrated into an SSC with a PVA/H_2_SO_4_ gel electrolyte to evaluate its electrochemical performance. Figure 7A shows the CV curves of the SSC at different scan rates (2–80 mV s^–1^), all exhibiting distorted rectangular shapes indicative of typical pseudocapacitance characteristics. Moreover, the CV curves maintained a consistent shape as the scan rate increased, demonstrating excellent stability and redox reversibility of the assembled device.^58^ The GCD curves (Figure 7B) displayed low IR drops and near triangular shapes at high current densities, indicating that the device showed excellent electrochemical reversibility and low resistance during charge–discharge cycles. Additionally, based on the GCD data, the specific capacitances of the device at various current densities were calculated to be 330, 317, 304, 210, and 140 F g^–1^ (Figure 7C). Figure 7D shows the Nyquist plot and equivalent circuit of the SSC. The corresponding fitted parameters are listed in Table 1, suggesting minimal resistance of the device, favoring charge transfer. Moreover, the steep slope of the straight line in the low-frequency region indicated the efficient ion-diffusion and charge-transfer capabilities of the SSC.

**Figure 7:**
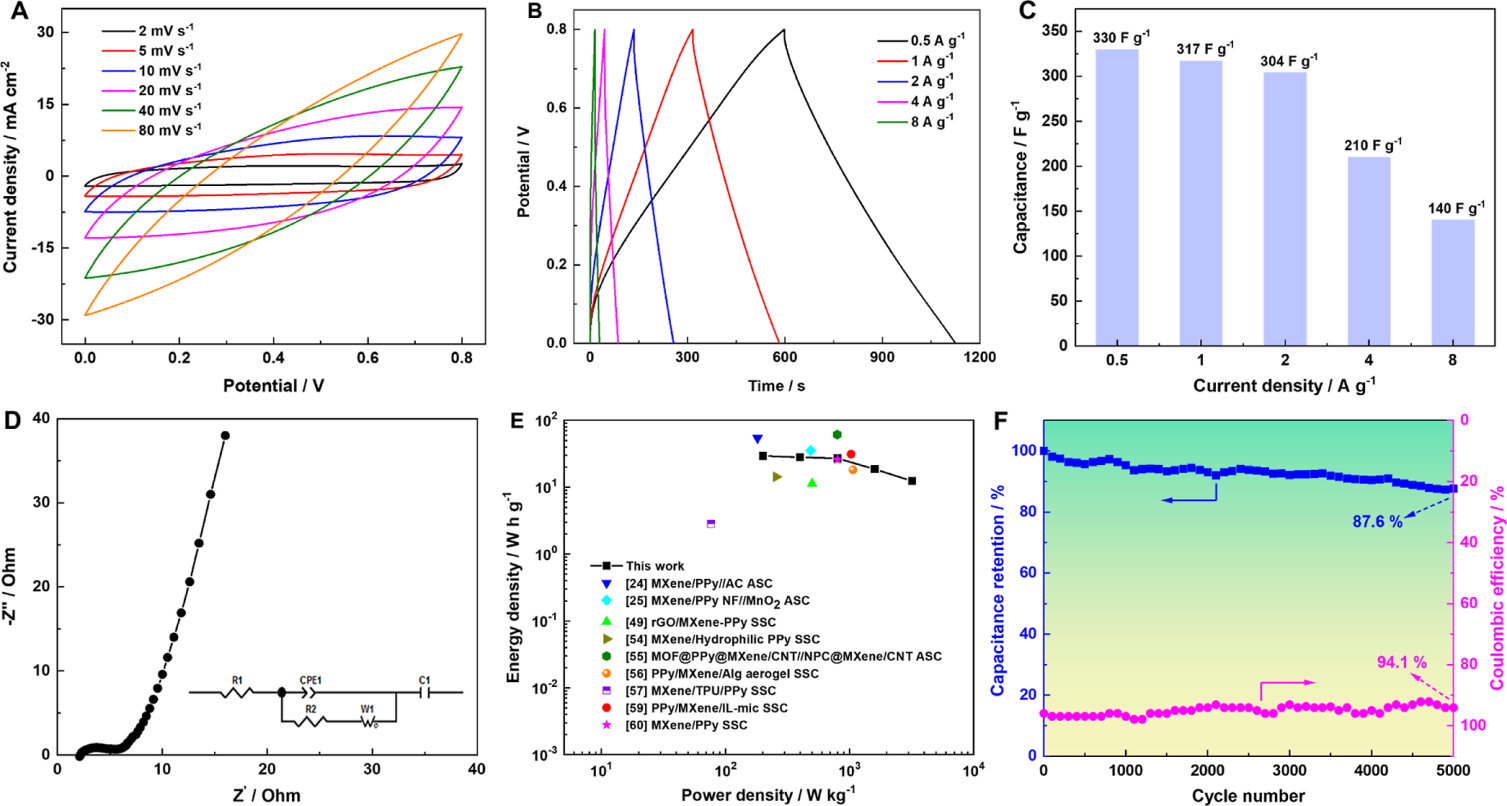
Electrochemical properties of the LPMX-2-based SSC device. (A) CV curves at 2–80 mV s^–1^, (B) GCD curves at 0.5–8 A g^–1^, (C) histogram of the relationship between the specific capacitance and current density, (D) Nyquist plots; inset: equivalent circuit, (E) Ragone plots of the device in comparison with recently reported supercapacitors based on different PPy/MXene composites, and (F) cycling stability and Coulombic efficiency at 2 A g^–1^.

The Ragone plot for the SSC (Figure 7E) shows an energy density of 29.3 W h kg^–1^ at a power density of 200 W kg^–1^, which is maintained at 27 W h kg^–1^ even at a higher power density of 800 W kg^–1^. The maximum power density reaches 3200 W kg^–1^. These results are comparable with those of recently reported PPy/MXene-composite-based supercapacitor devices.^24,25,49,54–57,59,60^ Interestingly, in the initial stages of increasing power density, the energy density of the device decreases only gradually. Figure 7F depicts the cycling stability and Coulombic efficiency of the LPMX-2-based SSC. After 5000 charge–discharge cycles at 2 A g^–1^, the device retains 87.6% of the initial capacitance and 94.1% of the initial Coulombic efficiency, indicating remarkable energy storage capacity and electrochemical reversibility. These features highlight the promising applications of the LPMX hybrid hydrogels in energy storage devices that require high energy and power densities simultaneously.

## 4 Conclusion

Herein, LS-modified PPy/MXene hybrid hydrogels with hierarchical porosity were fabricated through multiple self-assembly in situ polymerization processes. LS played several roles in the hydrogels: it simultaneously acted as a stabilizer, dopant, and cross-linker to optimize the PPy/MXene heterostructure and improved hydrogel electrochemical activity. The optimized LPMX-2 hydrogel exhibited excellent electrochemical performance, achieving a specific capacitance of 663.7 F g^–1^ at 0.2 A g^–1^ with exceptional cycling stability. An SSC assembled using LPMX-2 as the electrode material achieved energy densities of 29.3 and 12.4 W h kg^–1^ at power densities of 200 and 3200 W kg^–1^, respectively, while maintaining 87.6% capacitance retention and 94.1% Coulombic efficiency after 5000 charge–discharge cycles at 2 A g^–1^. These exceptional metrics resulted from the synergistic combination of electric double-layer capacitance from the MXene framework and pseudocapacitance from PPy nanoparticles within the hierarchical porous heterostructure. Owing to the aforementioned advantages, the hybrid hydrogel system emerges as a promising candidate system for advanced supercapacitor electrode materials that simultaneously require high energy and power densities.

## Supplementary Material



## References

[1] Zhang M. M., Xu S. X., Wang R. Y., Che Y. A., Han C. C., Feng W., Wang C. W., Zhao W. (2023). Electrospun nanofiber/hydrogel
composite materials and their tissue engineering applications. J. Mater. Sci. Technol..

[2] Thang N. H., Chien T. B., Cuong D. X. (2023). Polymer-based
hydrogels applied in
drug delivery: an overview. Gels.

[3] Wu Y. J., Parandoust A., Sheibani R., Kargaran F., Khorsandi Z., Liang Y. Y., Xia C. L., Van Le Q. (2023). Advances in gum-based
hydrogels and their environmental applications. Carbohyd. Polym..

[4] Qin C. L., Wu W. L., Gomaa H., Wu S., An C. H., Deng Q. B., Hu N. (2023). Hydrogel-based catalysts
for hydrogen
generation by the hydrolysis of B-H compounds under external physical
fields. J. Energy Chem..

[5] Li Y. N., Han Y. J., Li H. X., Niu X. H., Zhang D. Y., Wang K. J. (2024). Antimicrobial hydrogels: potential
materials for medical
application. Small.

[6] Ghosh S., Majhi J., Sharma S., Priya K., Bandyopadhyay A. (2023). A review on
the development of electron and ion conductive polymer hydrogels and
their composites for flexible and smart supercapacitors. J. Energy Storage.

[7] Sun Z., Ou Q., Dong C., Zhou J., Hu H., Li C., Huang Z. (2024). Conducting
polymer hydrogels based on supramolecular strategies for
wearable sensors. Exploration.

[8] Dou L. Y., Zhou S. X., Ma J., Zhao C., Cui P. X., Ye S. F., Feng P. Z., Gu X. Q., Huang S., Tao X. Y. (2024). Organic redox additive incorporated PANI hydrogel electrodes
for flexible high-energy-density supercapacitors. J. Mater. Chem. C.

[9] Devi L. S., Palathinkal R. P., Dasmahapatra A. K. (2024). Preparation of cross-linked PANI/PVA
conductive hydrogels for electrochemical energy storage and sensing
applications. Polymer.

[10] Li G., Huang K. X., Deng J., Guo M. X., Cai M. K., Zhang Y., Guo C. F. (2022). Highly conducting and stretchable
double-network hydrogel for soft bioelectronics. Adv. Mater..

[11] Roubert
Martinez S., Le Floch P., Liu J., Howe R. D. (2023). Pure conducting
polymer hydrogels increase signal-to-noise of cutaneous electrodes
by lowering skin interface impedance. Adv. Healthc.
Mater..

[12] Wang C. Y., Zhang J., Xu H., Huang C. H., Lu Y., Cui H. Y., Tan Y. B. (2022). Chitosan-driven biocompatible hydrogel
based on water-soluble polypyrrole for stable human-machine interfaces. Carbohyd. Polym..

[13] Sun Z. Y., Dong C., Chen B. D., Li W. B., Hu H. Y., Zhou J. S., Li C., Huang Z. D. (2023). Strong, tough, and
anti-swelling supramolecular conductive hydrogels for amphibious motion
sensors. Small.

[14] Zhang X. J., Zou W. J., Chen J. (2023). Effective
removal of Cr (VI) from
solution by three-dimensional polyaniline loaded composite porous
hydrogel. J. Mater. Sci..

[15] Kumar R., Barakat M. A. (2024). Flexible multifunctional chitosan/graphene
oxide/polyaniline
hydrogel thin films for adsorption of ibuprofen from aqueous solution. Cellulose.

[16] Liu J., Mckeon L., Garcia J., Pinilla S., Barwich S., Möbius M., Stamenov P., Coleman J. N., Nicolosi V. (2022). Additive manufacturing
of Ti_3_C_2_-MXene-functionalized conductive polymer
hydrogels for electromagnetic-interference shielding. Adv. Mater..

[17] Sarkar B., Li X. D., Quenneville E., Carignan L. P., Wu K., Cicoira F. (2021). Lightweight and flexible
conducting polymer sponges
and hydrogels for electromagnetic interference shielding. J. Mater. Chem. C.

[18] Sharma R.
K., Rastogi A. C., Desu S. B. (2008). Manganese oxide embedded polypyrrole
nanocomposites for electrochemical supercapacitor. Electrochim. Acta.

[19] Shi Y., Pan L. J., Liu B. R., Wang Y. Q., Cui Y., Bao Z. A., Yu G. H. (2014). Nanostructured
conductive polypyrrole
hydrogels as high-performance, flexible supercapacitor electrodes. J. Mater. Chem. A.

[20] Zhang Y., Xu A. Z., Yu Y., Ye S. Y., Zhao Z. Y., Cao W. F., Zhang S. Q., Qin Y. J. (2024). One-step fabrication
of integrated graphene/polypyrrole/carbon cloth films for supercapacitor
electrodes. Langmuir.

[21] Luo W. L., Ma Y., Li T. X., Thabet H. K., Hou C. P., Ibrahim M. M., El-Bahy S. M., Xu B. B., Guo Z. H. (2022). Overview of MXene/conducting
polymer composites for supercapacitors. J. Energy
Storage.

[22] Yi S., Wang L., Zhang X., Li C., Xu Y. A., Wang K., Sun X. Z., Ma Y. W. (2023). Recent
advances
in MXene-based nanocomposites for supercapacitors. Nanotechnology.

[23] Vigneshwaran J., Jose J., Thomas S., Gagliardi A., Narayan R. L., Jose S. P. (2024). PPy-PdO modified
MXene for flexible
binder-free electrodes for asymmetric supercapacitors: Insights from
experimental and DFT investigations. Chem. Eng.
J..

[24] Vigneshwaran J., Jose J., Thomas S., Gagliardi A., Thelakkat M., Jose S. P. (2022). Flexible quasi-solid-state
supercapacitors
based on Ti_3_C_2_-polypyrrole nanocomposites. Electrochim. Acta.

[25] Luo W. L., Sun Y., Han Y. Q., Ding J. X., Li T. X., Hou C. P., Ma Y. (2023). Flexible Ti_3_C_2_T_x_ MXene/polypyrrole
composite films for high-performance all-solid asymmetric supercapacitors. Electrochim. Acta.

[26] Zhang W., Ma J., Zhang W. J., Zhang P. G., He W., Chen J., Sun Z. M. (2020). A multidimensional
nanostructural design towards electrochemically
stable and mechanically strong hydrogel electrodes. Nanoscale.

[27] Qin Z., Zhao G., Zhang Y., Gu Z., Tang Y., Aladejana J. T., Ren J., Jiang Y., Guo Z., Peng X., Zhang X., Xu B. B., Chen T. (2023). A simple and
effective physical ball-milling strategy to prepare super-tough and
stretchable PVA@MXene@PPy hydrogel for flexible capacitive electronics. Small.

[28] Meng S. T., Liao P., Zhang X., Yan W. J., Qiu Z. H., Xu H. J. (2025). Ti_3_C_2_T_X_@PPy-reduced graphene oxide
heterostructure hydrogel for supercapacitor with excellent rate capability. J. Alloy. Compd..

[29] Milczarek G., Inganäs O. (2012). Renewable
cathode materials form biopolymer/conjugated
polymer interpenetrating networks. Science.

[30] Li F., Wang X., Sun R. (2017). A metal-free and flexible supercapacitor
based on redox-active lignosulfonate functionalized graphene hydrogels. J. Mater. Chem. A.

[31] Shen J., Cai M., Li G., Guo C. F., Qiu X., Qian Y. (2025). Lignosulfonate-derived
conducting organohydrogel as anisotrpic bioadhesive for motion-artifact-free
epidermal bioelectronics. Adv. Funct. Mater..

[32] Shao L., Qiu J. H., Feng H. X., Liu M. Z., Zhang G. H., An J. B., Gao C. M., Liu H. L. (2009). Structural investigation
of lignosulfonate doped polyaniline. Synth.
Met..

[33] Zhu L., Wu L., Sun Y., Li M., Xu J., Bai Z., Liang G., Liu L., Fang D., Xu W. (2014). Cotton fabrics
coated with lignosulfonate-doped polypyrrole for flexible supercapacitor
electrodes. RSC Adv..

[34] VahidMohammadi A., Moncada J., Chen H. Z., Kayali E., Orangi J., Carrero C. A., Beidaghi M. (2018). Thick and freestanding MXene/PANI
pseudocapacitive electrodes with ultrahigh specific capacitance. J. Mater. Chem. A.

[35] Wu W., Wang C., Zhao C., Wei D., Zhu J., Xu Y. (2020). Facile strategy of hollow polyaniline
nanotubes supported on Ti_3_C_2_-MXene nanosheets
for high-performance symmetric
supercapacitors. J. Colloid Interface Sci..

[36] Liu J., Wan M. X. (2001). Synthesis, characterization
and electrical properties
of microtubules of polypyrrole synthesized by a template-free method. J. Mater. Chem..

[37] Hou Z. Z., Lu H., Yang Q. H., Zhao Q. L., Liu J. (2018). Micromorphology-controlled
synthesis of polypyrrole films by using binary surfactant of Span80/OP10
via interfacial polymerization and their enhanced electrochemical
capacitance. Electrochim. Acta.

[38] Hou Z. Z., Yang Q. H., Lu H., Li Y. (2016). Towards enhanced electrochemical
capacitance with self-assembled synthesis of poly­(pyrrole-co-o-toluidine)
nanoparticles. J. Appl. Polym. Sci..

[39] Wei D., Wu W., Zhu J., Wang C., Zhao C., Wang L. (2020). A facile strategy
of polypyrrole nanospheres grown on Ti_3_C_2_-MXene
nanosheets as advanced supercapacitor electrodes. J. Electroanal. Chem..

[40] Wang Z. L., He X. J., Ye S. H., Tong Y. X., Li G. R. (2014). Design
of polypyrrole/polyaniline double-walled nanotube arrays for electrochemical
energy storage. ACS Appl. Mater. Interfaces.

[41] Fan Z. M., Cheng Z. J., Feng J. Y., Xie Z. M., Liu Y. Y., Wang Y. S. (2017). Ultrahigh volumetric performance of a free-standing
compact N-doped holey graphene/PANI slice for supercapacitors. J. Mater. Chem. A.

[42] Pan Z. H., Ji X. H. (2019). Facile synthesis
of nitrogen and oxygen co-doped C@Ti_3_C_2_ MXene
for high performance symmetric supercapacitors. J. Power Sources.

[43] Ma L., Zhao T., Xu F., You T., Zhang X. (2021). A dual utilization
strategy of lignosulfonate for MXene asymmetric supercapacitor with
high area energy density. Chem. Eng. J..

[44] Yang L., Lin F., Zabihi F., Yang S., Zhu M. (2021). High specific capacitance
cotton fiber electrode enhanced with PPy and MXene by in situ hybrid
polymerization. Int. J. Biol. Macromol..

[45] Jian X., He M., Chen L., Zhang M. M., Li R., Gao L. J., Fu F., Liang Z. H. (2019). Three-dimensional carambola-like MXene/polypyrrole
composite produced by one-step co-electrodeposition method for electrochemical
energy storage. Electrochim. Acta.

[46] Fu H., Yang Y., Chen Y., Zhou N., Dai J., Li D., Guo H., Qin Q., Zeng B., Yuan C., Xu Y., Dai L. (2025). Polyaniline-modified
NiCo layered double hydroxide
hollow nanocages for aqueous ammonium-ion supercapacitors. J. Colloid Interface Sci..

[47] Hu M., Hu T., Li Z., Yang Y., Cheng R., Yang J., Cui C., Wang X. (2018). Surface functional
groups and interlayer water determine
the electrochemical capacitance of Ti_3_C_2_T_x_ MXene. ACS Nano.

[48] Liang W., Zhitomirsky I. (2022). MXene-polypyrrole electrodes for asymmetric supercapacitors. Electrochim. Acta.

[49] Wang G. X., Jiang N. L., Xu Y. X., Zhang Z. X., Wang G. L., Cheng K. (2023). Solvent-assisted assembly
of reduced graphene oxide/MXene-polypyrrole
composite film for flexible supercapacitors. J. Colloid Interface Sci..

[50] Ronnasi B., Mahmoodian M., Mohammadi S., Yasoubi M., Sanaee Z. (2022). α-NSA
doped PPy @ Ti_3_C_2_T_x_ hybrid material
as a high-performance supercapacitor electrode. J. Mater. Res..

[51] Wang X., Wang X., Nian H., Chen T., Zhang L., Song S., Li J., Wang Y. (2022). Hierarchical MXene/polypyrrole-decorated
carbon nanofibers for asymmetrical capacitive deionization. ACS Appl. Mater. Interfaces.

[52] Li X. X., Xie H. D., Feng Y., Qu Y. Q., Zhai L. D., Sun H. H., Liu X. F., Hou C. P. (2023). All pseudocapacitive
MXene-PPy//MnO_2_ flexible asymmetric supercapacitor. J. Mater. Sci. Mater. Electron..

[53] Wang L., Wu H., Zhai X., Shi J., Zhou Q. Q., Li H., Wan J. M. (2023). Ti_3_C_2_T_x_ MXene/dopamine-modified
polypyrrole flexible composite electrodes with application in energy
storage devices. J. Alloy. Compd..

[54] Guo T. Z., Li X., Zhou H. W., Pang L. X., Zhou T., Shi Z. Q., Zhou D. (2024). Effect of large-sized
hydrophilic polypyrrole on electrochemical
properties of MXene films. J. Power Sources.

[55] Pathak I., Acharya D., Chhetri K., Chandra Lohani P., Hoon Ko T., Muthurasu A., Subedi S., Kim T., Saidin S., Dahal B., Yong Kim H. (2023). Ti_3_C_2_T_x_ MXene integrated
hollow carbon nanofibers with
polypyrrole layers for MOF-derived freestanding electrodes of flexible
asymmetric supercapacitors. Chem. Eng. J..

[56] Wu X. Y., Li J. Y., Zhu Y. F., Cai W. R., Xu J., Qin Y., Kong Y. (2023). Porous polypyrrole/Ti_3_C_2_T_x_/alginate aerogels prepared by sacrificial
template strategy
as electrode materials in symmetric supercapacitors. J. Energy Storage.

[57] Zhang J., Wang X. C., Hang G. G., Zhang W. J., Zheng Z. F., Duan J., Liu Z. (2024). Polypyrrole
in-situ polymerized MXene/TPU
fiber electrode for flexible supercapacitors. Compos. Commun..

[58] Fu, H. ; Chen, Y. ; Yang, Y. ; Zhou, N. ; Dai, J. ; Li, D. ; Luo, Q. ; Wang, X. ; Jia, R. ; Ren, H. ; Qin, Q. ; Xu, Y. ; Dai, L. Polyaniline interface engineering-enabled oxygen vacancy-enriched NiCo-LDHs for ammonium-ion supercapacitors. Inorg. Chem. Front. 2025, Advance online publication. 10.1039/D5QI00083A

[59] Fan Q., Zhao R. Z., Yi M. J., Qi P., Chai C. X., Ying H., Hao J. C. (2022). Ti_3_C_2_-MXene
composite films functionalized with polypyrrole and ionic liquid-based
microemulsion particles for supercapacitor applications. Chem. Eng. J..

[60] Varghese S. M., Mohan V. V., Suresh S., Bhoje Gowd E., Rakhi R. B. (2024). Synergistically modified Ti_3_C_2_T_x_ MXene conducting polymer nanocomposites
as efficient
electrode materials for supercapacitors. J.
Alloy. Compd..

