# Supplementary material for: Toward Superior Electrochemical Capacitance with Hierarchically Nanostructured Polypyrrole/MXene Hybrid Hydrogel Modified by Lignosulfonate
Source: ACS Omega. 2025 Jun 30;10(27):29476–87. doi: 10.1021/acsomega.5c02827 (PMC12268449; doi:10.1021/acsomega.5c02827)
Supplement: Supplementary file 1 [file ao5c02827_si_001.pdf]

# Supporting Information

## Towards Superior Electrochemical capacitance with Hierarchically Nanostructured Polypyrrole/MXene Hybrid Hydrogel Modified by Lignosulfonate

Zhenzhong Hou *, Qinghao Yang, Hai Lu, Ying Li, QiuLi Zhao

*College of Materials Science and Engineering, Xi'an University of Science and Technology, Xi'an 710054, China*

* *Correspondence to: Z. Hou (E-mail: hzzhong1981@yeah.net)*

Table S1 Detailed compositions and conductivities of the LPMX samples

| Sample | Mass ratio of MXene to pyrrole / % | Mass ratio of LS to pyrrole / % | Conductivity / S $cm^{-1}$ |
|---|---|---|---|
| LPMX-1 | 3.4 | 5 | 15.6 |
| LPMX-2 | 3.4 | 10 | 36.8 |
| LPMX-3 | 3.4 | 15 | 9.1 |
| LPMX-4 | 3.4 | 20 | 2.46 |

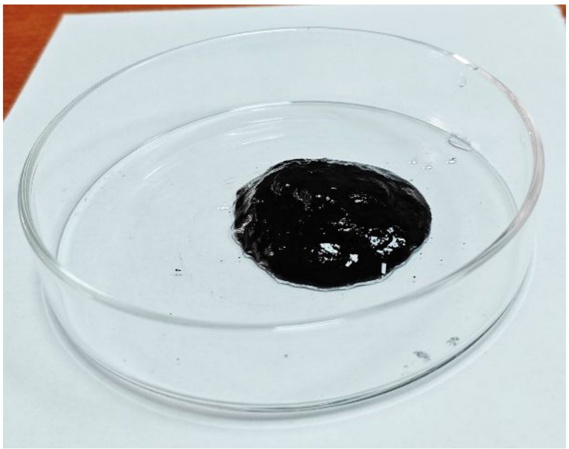

Figure S1 Macroscopic image of the as-prepared LPMX hybrid hydrogel.

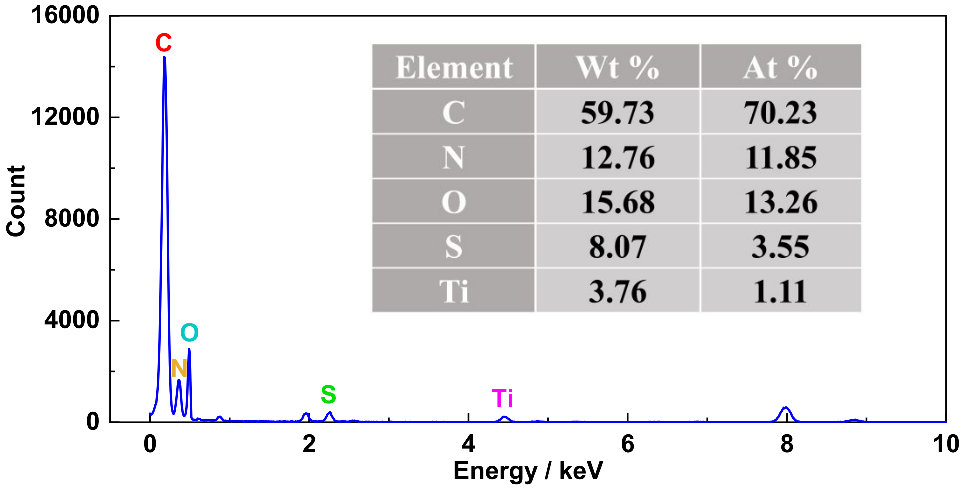


| Element | Wt % | At % |
|---|---|---|
| C | 59.73 | 70.23 |
| N | 12.76 | 11.85 |
| O | 15.68 | 13.26 |
| S | 8.07 | 3.55 |
| Ti | 3.76 | 1.11 |

Figure S2 TEM EDS pattern and elements ratios of the LPMX-2.

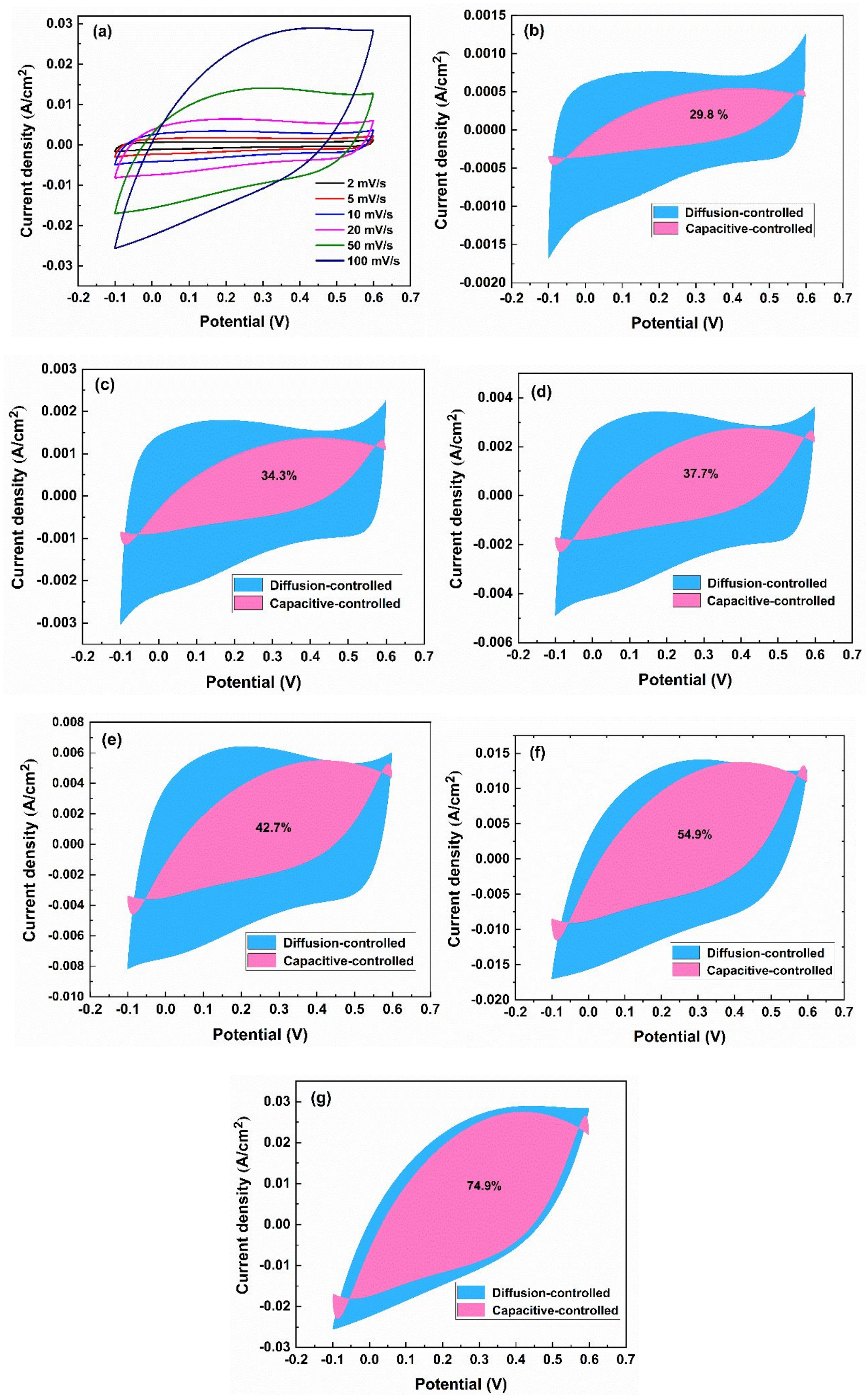


Figure S3 (a) CV curves of the LPMX-2 electrode at varying scan rates from 2 to 100 mV $s^{-1}$. (b-g) CV profiles of the LPMX-2 electrode at scan rates of 2-100 mV $s^{-1}$ showing the capacitive contribution (magenta region) to the total current.

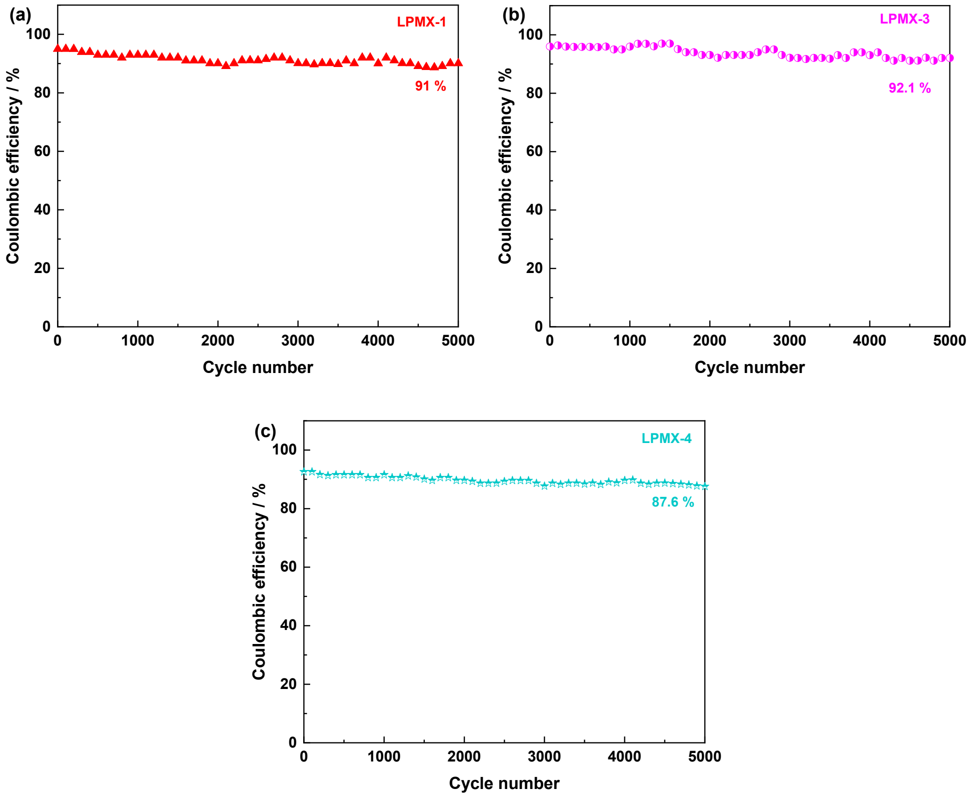


Figure S4 Coulombic efficiency of (a) LPMX-1, (b) LPMX-3 and (c) LPMX-4 after 5000 cycles at 2 A $g^{-1}$.
